# On Uterine Compression as a Means of Completing Delivery

**Published:** 1871

**Authors:** G. Chantreuil

**Affiliations:** Paris


### Article 214.—*On Uterine Compression as a Means of Completing Delivery.* By Dr. G. Chantreuil, of Paris.

[Archives Generales de Medicine, No. 10, 1871.]

"The method of uterine compression is simple in principle and easy in execution. Its aim being to strengthen the uterine contractions, it ought to be applied during the pains and not in the intervals; the sooner one operates after the expulsion of the fœtus, the more rapid is the success of the proceeding; one may, however, succeed a quarter or half an hour after the expulsion of the fœtus, but these conditions are most unfavorable.

"When the contraction of the uterus has attained its maximum during the first pain which is normally manifested after the issue of the infant, the medical attendant grasps with the whole hand the fundus of the viscus in such a manner that the fundus and the upper part of the anterior surface of the womb are under the palm of the right hand placed transversely. Maintained pressure from above downwards, and from before backwards, is then kept up by this hand, its action being increased by the support of the dorsal surface of the left hand. With this compression the placenta and membranes are detached, and then pass in a close mass through the orifice of the uterus; sometimes they pass all at once from the external genitals like a cherry-stone seized between the thumb and index-finger.

"When I undertook the duties of interne to the maternity department of the Cochin Hospital, M. de St. Germain permitted me to practice uterine compression, and himself had recourse to

*Dr. Althaus "On the Lithia Springs of Baden-Baden," *Medical Times and Gazette*, vol. ii., 1861.

† *Essentials of Materia Medica*, 3d edit., p. 105, 1868.

this proceeding, with very good results, whenever he was in the lying-in wards during a labor. My successor, two internes and two midwives of the establishment followed our example, and experienced no difficulty in the performance of this operative proceeding, by means of which they always succeeded in bringing about complete expulsion of the placenta and the membranes.

"In five hundred and forty observations collected by myself, I have been able to show a tolerably rapid expulsion of the placenta and membranes. This expulsion takes place sometimes at the time of the first contraction following the issue of the fœtus, but more frequently with the second and third contractions.

"In a table prepared by myself, for showing the different epochs at which expulsion of the placenta occurred in the numerous labors in which uterine compression was practiced, it may be seen that delivery was completed during the first six minutes, and even during the first three minutes that elapsed after the issue of the fœtus.

"In eleven cases the expulsion of the placenta was delayed for ten minutes, in six for fifteen minutes, and in three other cases for twenty minutes. With regard to the three last cases, I find the following facts recorded:

"Case 1.—Child born, but placenta not expelled at the time of admission. Here the uterine compression was not employed immediately after the birth.

"Case 2.—One of a woman in whom contractions were very slight after the birth of the child.

"Case 3.—In this case the contractions were weak and irregular during labor.

"In cases where the contractions, particularly those of the expulsive period, are short, feeble and irregular, completion of the delivery by uterine compression is brought about less rapidly. On the contrary, when the expulsive pains have been strong and prolonged through the last period of labor, the issue of the placenta is almost immediate.

"In cases of premature labor, this method seems to act less actively. The weight of the placenta has no apparently sensible influence upon the rapidity of the result. According to Crede, the woman suffers no inconvenience from the compression, beyond a sharp pain at the moment of the manœuvre, altogether comparable to that produced by a strong pain. But there is a great difference between this phenomenon and the painful distension of the genital organs, which must be produced when the placenta has to be sought for in the uterine cavity. For my own part, I have observed some time after delivery some tenderness of the womb, which soon disappeared either spontaneously or under the influence of opiated cataplasms, and never in advanced metritis.

"It is evident that by this proceeding one avoids rupture of the cord and its consequences, retention of the placenta, purulent infection, &c. Deviation and inversion of the uterus cannot take place, since the umbilical cord is not dragged upon. The regular and rapid contraction of the uterus, which succeeds the rapid expulsion of the placenta, prevents hæmorrhage. In the cases which I have collected from the practice of the Cochin maternity, there is not a single instance of hæmorrhage occurring during or after the expulsion of the placenta. Other observers, as Crede, Clarke, Speigelberg and Meyer, assert that they have not observed hæmorrhage since they have employed exclusively this proceeding in their practice. During this epoch, also, they state that they have not seen those cases which are described in obstetric works, under the designations of adhesion of the placenta, and of retention and inclusion of this organ, and seem disposed to inquire whether such cases really exist. It would seem, in fact, that the instances which have been described as such are due simply to failure of energy in the contractions; by strengthening these contractions the placenta, which is neither adherent nor inclosed, is detached and expelled. M. Depaul, in his clinical lectures, does not readily admit that the superior portion of the body of the uterus may separately contract to form a cavity to which the placenta can be retained. According to this authority, it would be more conformable to practice to explain the hour-glass contraction taken by the womb in certain cases by contraction of the internal orifice; the part below the contraction could not be anything else than the enlarged and dilated cavity of the neck; the superior part would be constituted by the whole body of the organ, inclosing in its cavity the placenta and membranes rendered sluggish by failure of the expulsive forces.

"It may be objected that the dangers of this method, when actually adopted, are due particularly to the inexact application of the practical means which constitute it, and that one ought not to put to its account the inconvenience resulting from the ignorance of persons who employ it.

"I would acknowledge in part the justice of this remark, but one must agree that at the stage of delivery, when the cord is pulled down, there is some vague and uncertain practice which is left to the sagacity and experience of the accoucheur. He does not know at what precise period he ought to exercise traction, what is the force which he ought to employ, and what, above all, is the superior limit of this force compatible with the health of the woman.

"There exists, it is true, this good means of avoiding a mistake—not to pull upon the cord too soon—that is to say, to examine the woman, and to make out whether the placenta is engaged in the neck of the womb. But, in order to make this diagnosis, it is necessary to have a certain skill in touch which, unfortunately, is not very readily acquired. Besides, in a great number of cases, we have observed this precaution to be entirely neglected before traction has been made on the cord.

"With regard to midwives, especially, I think that it is useful to lay down fixed rules for the removal of the placenta, in order that their minds may be more calm and their conduct less uncertain during the period following accouchement. With the method of uterine compression, this uncertainty disappears. Pupils when impressed with the rule of never to draw down the cord will be led to avoid the dangers of breaking this structure, of producing uterine invasion, and of tearing the placenta; on the other hand, with the actually current ideas on this subject, the most prominent idea in the mind of the pupil, who has devoted very much time to the study of obstetrics, is *traction on the umbilical cord.*

"The very variable epoch at which these tractions ought to be made, the important precaution which ought to be taken before carrying out this practice, and which consists in determining whether the placenta is already engaged in the orifice, are details which may be readily forgotten, and the best proof of this fact is that the traction is often made too soon or too late; the consequences of a premature intervention are too well known—rupture of the cord, uterine invasion, &c. When the traction is made too late, we find contraction of the internal orifice, inclusion of the placenta in the uterine cavity, &c.

"These accidents, then, will be remedied by persuading practitioners not to touch the cord, but to replace the force traction by the *vis a tergo.*

"These external manœuvres are much less repugnant to midwives and to practitioners but little familiar with obstetrics than the operation, which consists in introducing the whole hand into the uterine cavity for the purpose of seeking the placenta. This is what happens, particularly in cases of rupture of the cord; the attendant hesitates, perhaps with reason, to perform without delay, the necessary internal manœuvres; he waits two or three hours, or even longer, relying upon the efforts of nature. If these are powerless, recourse is then had to a more experienced or a bolder *confrere,* who meets with, at the time of his arrival, excessive and even insurmountable difficulties.

"Whilst speaking of the advantages of uterine compression, it may be stated that the generalization of the employment of this method would result in a more active attention to the latter stages of delivery from competent persons; it would prevent this part of the process from being confided to the care of females, altogether strangers to the obstetric art, and attention would be directed by the necessity of exercising a strict control over the state of the uterus after the expulsion of the fœtus. The uterine contractions ought to be excited in order for the expulsion of the placenta to take place spontaneously. If these conditions be fulfilled, it may be readily comprehended that hæmorrhage will rarely occur.

"I am well aware that in all treaties on obstetrics it is recommended to keep a watch over the uterus after expulsion of the fœtus, in the fear of inertia and a distension of this organ from internal loss; it is evident that this recommendation is often forgotten in practice.

"Midwives, and sometimes practitioners, after cutting and tying the cord, leave the mother to attend to the child, returning after an interval to complete the delivery; they pull at the cord, and if there is much resistence, leave it; some time having elapsed, they again pull, until the placenta arrives at the vulva. It may be conceived how accidents may result from this mode of proceeding; abundant and rapid hæmorrhage may occur whilst the midwife is occupied in cleansing and wiping the infant.

"The employment of uterine compression would do away with this custom, for, according to the rule laid down by Crede, the hand of the accoucheur ought to be applied to the uterus immediately after the birth of the child, and be kept there until the expulsion of the placenta.

"To resume, I think that the method of completing delivery, called uterine compression, presents great advantages. By nature we are not enthusiastic about new things, but I hold that it is the duty of every medical man to make known what he may think to be useful in the practice of his art. I hope that no one will pronounce against this mode of completing delivery without having employed it, and I await the results of my *confreres'* experiences on this subject."

